# Highly Efficient CRISPR/Cas9-Mediated Homologous Recombination Promotes the Rapid Generation of Bacterial Artificial Chromosomes of Pseudorabies Virus

**DOI:** 10.3389/fmicb.2016.02110

**Published:** 2016-12-23

**Authors:** Jin-Chao Guo, Yan-Dong Tang, Kuan Zhao, Tong-Yun Wang, Ji-Ting Liu, Jia-Cong Gao, Xiao-Bo Chang, Hong-Yu Cui, Zhi-Jun Tian, Xue-Hui Cai, Tong-Qing An

**Affiliations:** State Key Laboratory of Veterinary Biotechnology, Harbin Veterinary Research Institute, Chinese Academy of Agricultural SciencesHarbin, China

**Keywords:** CRISPR/Cas9, homologous recombination, bacterial artificial chromosome, pseudorabies virus, knock-in

## Abstract

Bacterial artificial chromosomes (BACs) are powerful tools for the manipulation of the large genomes of DNA viruses, such as herpesviruses. However, the methods currently used to construct the recombinant viruses, an important intermediate link in the generation of BACs, involve the laborious process of multiple plaque purifications. Moreover, some fastidious viruses may be lost or damaged during these processes, making it impossible to generate BACs from these large-genome DNA viruses. Here, we introduce the CRISPR/Cas9 as a site-specific gene knock-in instrument that promotes the homologs recombination of a linearized transfer vector and the Pseudorabies virus genome through double incisions. The efficiency of recombination is as high as 86%. To our knowledge, this is the highest efficiency ever reported for Pseudorabies virus recombination. We also demonstrate that the positions and distances of the CRISPR/Cas9 single guide RNAs from the homology arms correlate with the efficiency of homologous recombination. Our work show a simple and fast cloning method of BACs with large genome inserted by greatly enhancing the HR efficiencies through CRISPR/Cas9-mediated homology-directed repair mechanism, and this method could be of helpful for manipulating large DNA viruses, and will provide a successful model for insertion of large DNA fragments into other viruses.

## Introduction

Pseudorabies virus (PRV), the causative agent of Aujeszky's disease, is a member of the family *Herpesviridae*, the subfamily *Alphaherpesvirinae*, and the genus *Varicellovirus* (Matthews, 1982). PRV can infect most mammals, except humans and other higher primates, but pigs are the only known natural reservoir (Klupp et al., 1995). Because it is a neurotropic pathogen, effectively invading the peripheral nervous system and establishing a lifelong latent infection in the neurons resident in the peripheral ganglia (Smith, 2012), it is an important model to analyze the virus transportation mechanism in the nerve system. A PRV variant emerged in China in 2011 that caused typical neurological symptoms and high mortality in newborn piglets on many farms, resulting in substantial economic losses (An et al., 2013).

Bacterial artificial chromosomes (BACs) are especially useful platforms for studying herpesviruses because the genomes of these viruses are too large to be cloned into plasmids or cosmids (Wagner et al., 2002). Since the first generation of BACs containing mutant murine cytomegalovirus (Messerle et al., 1997), a variety of BACs containing different viruses have been generated, including human cytomegalovirus using homologous recombination (HR) (Borst et al., 1999), varicella zoster virus with overlapping cosmid inserts (Tischer et al., 2007), Human herpesvirus 6A using the direct ligation method (Borenstein and Frenkel, 2009), and herpesvirus using *in vitro* transposition (Zhou et al., 2009). A herpesvirus genome is normally cloned into a BAC with HR between the viral genome and the flanking DNA fragments of the BAC cassette (Adler et al., 2003). Because the efficiency of HR is low, the recombinant virus have to subject to multiple rounds of plaque purification, which is labor intensive and time consuming, especially for some slow-growing viruses. More importantly, for some fastidious viruses that fail to produce plaques in cells, such as Kaposi's sarcoma-associated herpesvirus, it can be impossible to isolate the purified recombinant virus (Zhou et al., 2002).

The efficacy of HR can be significantly improved by DNA double-stranded breaks (DSBs) (Gao et al., 2016). DSBs have been shown to stimulate cell repair pathways, including error-prone nonhomologs end joining (Bibikova et al., 2003) and homology-directed repair (Urnov et al., 2005). Homology-directed repair can precisely repair the damaged DNA in the presence of homologous donor DNA, whereas nonhomologs end joining is an error-prone mechanism that always results in a heterogeneous pool of insertions and deletions (Ran et al., 2013). CRISPR/Cas9 is emerging as a powerful tool for DNA engineering in diverse organisms, and allows efficient DNA editing (Cong et al., 2013). Gene knock-in of large DNA viruses with CRISPR/Cas9 has been reported, including of adenovirus (Bi et al., 2014), Herpes simplex virus *1* (Bi et al., 2014), PRV (Xu et al., 2015; Liang et al., 2016; Tang et al., 2016), and Epstein–Barr virus (Kanda et al., 2016). However, there is little information about the features of CRISPR/Cas9 that are important in enhancing the efficiency of the HR between PRV and BAC. In this study, we systematically studied the correlation between the location of single guide RNA (sgRNA) and the efficacy of HR in the construction of a BAC encoding PRV.

## Materials and methods

### Virus and cell line

The PRV-HLJ8 strain (GenBank accession no. KT824771) isolated in 2014 was replicated. Vero cells were cultured in Dulbecco's modified Eagle's medium (Gibco, Grand Island, NY, USA). All culture media were supplemented with 10% heat-inactivated fetal bovine serum (Gibco Life Technologies).

### Generation and linearization of the transfer vector pBAC-GFP62

The plasmid pBeloBAC11 vector (New England Biolabs) was first inserted into two loxP sites (ATAACTTCGTATAATGTATGCTATACGAAGTTAT) to generate pBeloBACloxP. The green fluorescence protein (GFP) cassette was cut from the pEFGP-N1 vector (Clontech) with *Bam*HI and *Hin*dIII restriction enzymes and inserted into the pBACloxp11 vector, generating pBAC-GFP. The upstream homologous arm (including the entire US6 gene and partial US4 gene, 1695 bp) and the downstream homologous arm (including the partial US2 gene and partial US1 gene, 1547 bp) were amplified with PCR. They were then inserted into pBAC-GFP, constructing the transfer vector pBAC-GFP62 (Us4-Us6, position −10648 to −8946 bp; Us2-Us1, position 402 to 1957 bp). After pBAC-GFP62 was confirmed with enzymatic digestion and DNA sequencing, the plasmid was linearized with *Swa*I and purified with the QIAEX II Gel Extraction Kit (Qiagen). The primers used are described in Table 1.

**Table 1:** **Sequences of PCR primers and sgRNA oligonucleotides**.

**Fragments**	**Name**	**Sequences (5′–3′)**	**Length (bp)**
Us4-Us6	Us4F	CTGATTTAAATTTCGTCTCGCCCTCTGACATC	1695
	Us6R	CTGTTAATTAACCCCCTCAGGCGGAAGAAGAT	
Us2-Us1	Us2F	TGCATCGATCATCACCACCGAGACGCACGA	1547
	Us1R	CCGATTTAAATGGACGGGGACGACTTTGACGG	
CMV-GFP	CMV-GFP-F	TATGGATCCTGATCGATCGATTTAAATCGTTAATTAATAGTTATTAATAGTAATCAATTAC	1500
	CMV-GFP-R	CGCAAGCTTACATTGATGAGTTTGGACAAACCAC	
gB	gB-F	GTCACCTTGTGGTTGTTG	180
	gB-R	CCACATCTACTACAAGAACG	
gC	gC-F	TCTCGGTGGCCGTCAAGGG	740
	gC-R	GCGGACCTCGAAGGTCTCCC	
gG	gG-F	ACCGCTACGACACCAAGGTC	706
	gG-R	GCCGCCGTCAAAGAACCAG	
gH	gH-F	AGCTCCAGGACACCCTCTTCGG	730
	gH-R	GGGCGCTGCACAAAGTACCAC	
gK	gK-F	GCACGTCCCACAGGTAGGCG	498
	gK-R	CCCGACTGGGTGCTCTTCC	
gN	gN-F	TACAATCGCCTGCACCTCGC	765
	gN-R	AGGAGCCGTGGCCATCGTAG	
gI	gI-F	TGCTGAACGCCAGCGTCGTGT	150
	gI-R	GCCGGGCCACGCAGGCGATCC	
gE	gE-F	CATCTGGCTCTGCGTGCTGTGCTCC	367
	gE-R	GGTCACGCCATAGTTGGGTCCATTCGT	
sgRNA-Us7	sgRNA-Us7-R	CACCGGTCGGGGGCGTCCTCTTCAG	
	sgRNA-Us7-F	AAACCTGAAGAGGACGCCCCCGACC	
sgRNA-Us8	sgRNA-Us8-R	CACCGGGGCAGGAACGTCCAGATCC	
	sgRNA-Us8-F	AAACGGATCTGGACGTTCCTGCCCC	
sgRNA-Us9	sgRNA-Us9-F	CACCGCGACGTCCTGCTGGCCCCCA	
	sgRNA-Us9-R	AAACTGGGGGCCAGCAGGACGTCGC	
sgRNA-Us2	sgRNA-Us2-F	CACCGACCGTGGTCACGCTGATGGA	
	sgRNA-Us2-R	AAACTCCATCAGCGTGACCACGGTC	

### Cotransfection and plaque purification assay

The PRV HLJ genome was extracted as previously described (Szpara et al., 2011). For all cotransfections, 3 μg of the PRV HLJ genome and 3 μg of linearized pBAC-GFP62 were added to each individual well. For control group, 2 μg of pCas9 vector (pX330) was cotranfected; for single incision group, each was cotransfected with 1 μg of pCas9-sgRNA and 1 μg of pCas9 vector, respectively; for double-incision group, each individual well was cotransfected with 2 μg of pCas9-sgRNAs (two pCas9-sgRNAs, each 1 μg). To all the solutions was added 16 μL of X-tremeGENE HP DNA Transfection Reagent (Roche, Basel, Switzerland), according to the manufacturer's instructions. After 72 h, the cells were collected and subjected to three freeze–thaw cycles. The recombinant virus BAC-HLJ (vBAC-HLJ) was purified with plaque purification in Vero cells overlain with 1% low-melting-point agarose and Dulbecco's modified Eagle's medium containing 2% fetal bovine serum. After seven rounds of purification, all the plaques expressed GFP, were detected with fluorescence microscopy.

### Electrotransformation assay

The replicative intermediate (covalently closed circular) PRV HLJ DNA was isolated as previously described (Hirt, 1967). *Escherichia coli* DH10B cells were electroporated with the circular genome of PRV (2.5 kV, 200 Ω, 25 μF; Bio-Rad) (Mahony et al., 2002), and the cells were then incubated in 1 mL of SOC medium for 1 h at 37°C with shaking at 220 rpm. The medium was centrifuged, plated on Luria–Bertani (LB) plates containing 17 μg/mL chloramphenicol, and incubated for 24 h at 37°C. Chloramphenicol-resistant colonies were inoculated into 5 mL of LB broth containing 17 μg/mL chloramphenicol and grown at 37°C for 16 h.

### Identification of pBAC-HLJ with PCR

To confirm the integrity of the PRV HLJ genome inserted into the BAC, genes spread throughout the pBAC-HLJ genome were detected with PCR, including the partial BAC sequence, GFP in the transfer vector pBAC-GFP62, and essential genes in the PRV genome: gB, gC, gG, gH, gK, and gN. The genes deleted from pBAC-HLJ, gE and gI, were also identified in the parental virus, PRV HLJ, as a positive control.

### Pulse-field gel electrophoresis (PFGE)

The DNA (5 μg) of wild-type PRV HLJ, the pBAC-HLJ plasmid, or rescue virus BAC-HLJ (resBAC-HLJ) was digested with *Bam*HI for 3 h at 37°C. The three samples were analyzed with PFGE, with an upper limit of 60 kb and a lower limit 1 kb, with a previously reported method (Han et al., 2013).

### Electron microscopy

Vero cells were infected with resBAC-HLJ and harvested at 48 h postinfection (hpi), after three rounds of freeze–thaw. Cell culture medium containing resBAC-HLJ was first centrifuged at 3000 g for 10 min, and then the supernatant was centrifuged at 10,000 g for 10 min. The sample was negatively stained with 2% phosphotungstic acid, and the morphology of the viral particles was examined with transmission electron microscope (Hitachi).

### Replication kinetics of rescued vBAC-HLJ

Vero cells were infected with PRV HLJ and resBAC-HLJ at 10^3^ 50% tissue culture infective doses (TCID_50_), and were harvested at 12, 24, 36, 48, 60, and 72 hpi. After the inoculated cells were freeze thawed three times, the cellular supernatants were collected and serially diluted 10-fold from 10^−1^ to 10^−9^. Vero cells in 96-well plates were infected with 0.1 mL/well of serially diluted cell supernatant, in sextuple. Five days after infection, TCID_50_ was determined with the Reed–Muench method. All data are shown as the means of three independent experiments.

### CRISPR/Cas9 sgRNA incision efficacy assay

The sgRNAs efficacies were evaluated as described by Tang et al. (2016). In brief, different sgRNAs of CRISPR/Cas9 were constructed and then were transfected into Vero cells. Twelve hours post-transfection, cells were infected with 0.01 MOI of Luciferase Tagged PRV. Twelve hours post infection, luciferase activity was evaluated.

### HR efficiency assay

The cell inoculums were collected 72 h after cotransfection, and subjected to three freeze–thaw cycles. The numbers of recombinant viruses were calculated by counting the GFP-stained colonies under an inverted fluorescence microscope and counting the total viruses with a crystal violet assay. The efficiency of HR was determined from the plaque assay as the ratio of recombinant viruses to total viruses.

### Statistical analysis

The data were analyzed with one-way analysis of variance followed by Tukey's multiple comparison test. *P*-values of 0.05 or less were considered statistically significant.

## Results

### Generation of BAC of PRV

**Figure 1:**
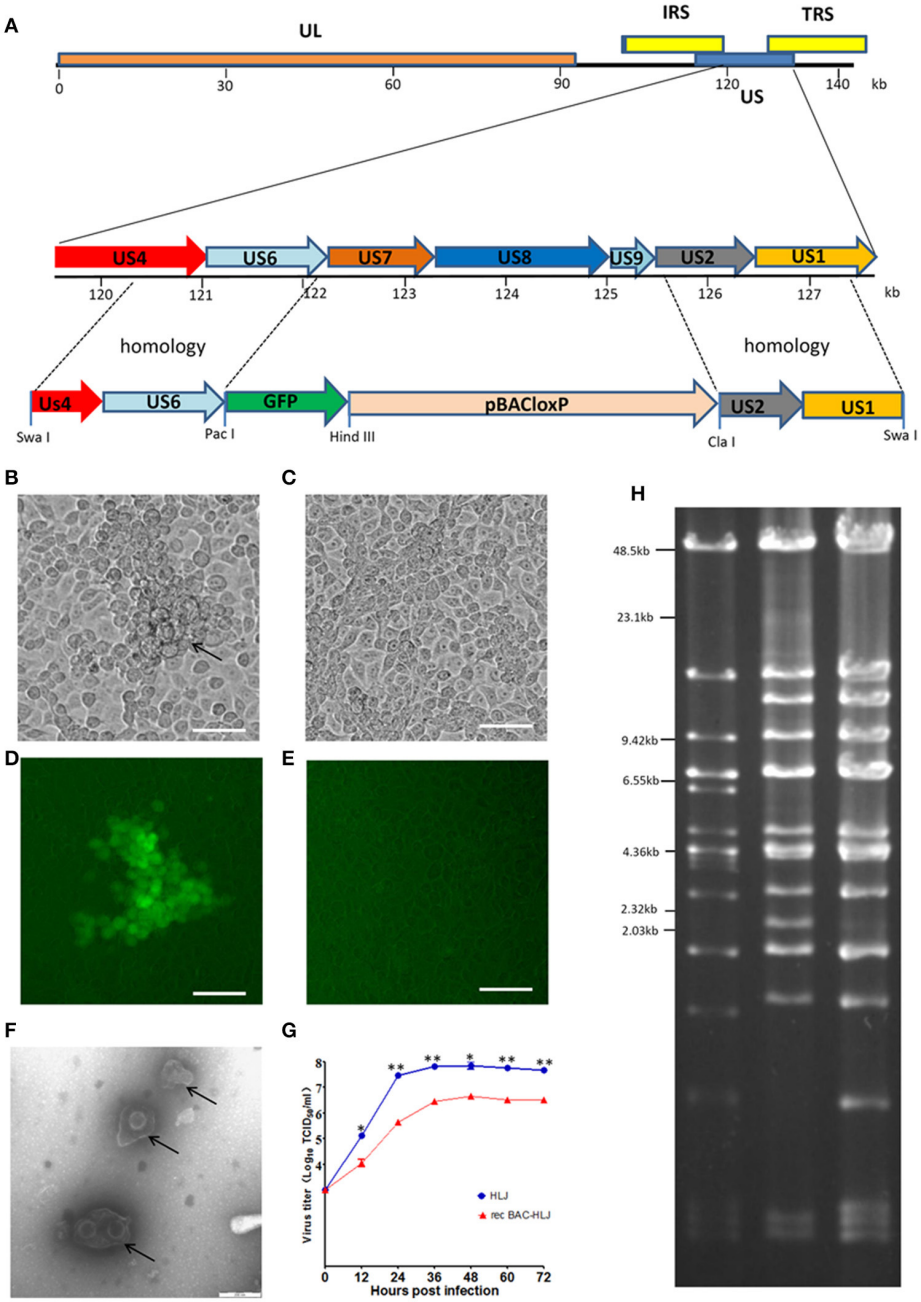
**Construction and identification of PRV BAC. (A)** Construction of the recombinant virus and insertion of the pBAC-GFP62 vector into the viral genome are illustrated. The genome of PRV HLJ is illustrated at the top, with a portion of the right end of the genome expanded to show the Us4, Us6, Us7, Us8, Us9, Us2, and Us1 genes. All recombinants were used to transfect Vero cells. Electrotransformation of the circular BAC-HLJ genome yielded the full-length clone pBAC-HLJ. **(B–E)** resBAC-HLJ identification. **(B,D)** show the CPE and GFP of resBAC-HLJ; **(C,E)** show the control. Bar: 100 μm. **(F)** Scanning electron microscopy. Viral particles with apparent PRV morphology were detected. **(G)** Replication kinetics of resBAC-HLJ compared with those of wild-type PRV HLJ. Vero cells in six-well plates were infected with 1000 TCID_50_ of either PRV HLJ or resBAC-HLJ. The culture supernatants were recovered at 12, 24, 36, 48, 60, and 72 hpi, and TCID_50_ was determined for each. Titrations were performed in triplicate; error bars represent standard errors of the mean. **(H)** *Bam*HI digestion. Pulse-field gel electrophoresis (PFGE) assay established the restriction enzyme profiles of PRV HLJ, pBAC-HLJ, and resBAC-HLJ in 1% agarose. DNA fragments were stained with ethidium bromide and photographed. ^*^P < 0.05, ^**^P < 0.01.

A graphical abstract of the experiment was illustrated (Figure S1), to have a clear knowledge of the procedures to generate pBAC-HLJ. First the transfer vector pBAC-GFP62 was constructed (Figure 1A). After pBAC-GFP62 was confirmed with enzymatic digestion and DNA sequencing, the plasmid was linearized and purified. Vero cells were cotransfected with pBAC-GFP62 and the extracted PRV HLJ genome DNA (Szpara et al., 2011), and the HR efficiency of the group adding additional 2 μg pCas9 vector was regarded as the control. An equal amount of pCas9-Us8 was added to the plasmid and PRV HLJ genome, together with the transfection reagent, to enhance the HR efficiency. At 72 h post-transfection, the HR efficiencies of the control and the Us8 were calculated. The HR efficiency of CRISPR/Cas9 was higher than that of the control. After recombinant viruses displaying GFP fluorescence were detected, seven rounds of plaque purifications were performed, and the pure recombinant virus was identified by testing for glycoprotein E (gE, Us8) and gI (Us7) with PCR. The vBAC-HLJ was propagated and the circular intermediates were isolated with Hirt extraction (Hirt, 1967). *Escherichia coli* strain DH10B was electroporated with the circular viral genome, as described previously (Mahony et al., 2002), and chloramphenicol-resistant clones were assayed for the successful replication of pBAC-HLJ.

After pBAC-HLJ was confirmed with PCR (Figure S2), it was used to transfect Vero cells to rescue the virus. The pBAC-HLJ-transfected cells were checked for a cytopathic effect (CPE) and green (GFP) fluorescence (Figures 1B,D). The Vero cells without pBAC-HLJ transfected were detected as controls (Figures 1C,E). We observed the resBAC-HLJ particles with electron microscopy, and their morphology, with an apparently external envelope, is shown in Figure 1F.

The replication kinetics of resBAC-HLJ were determined, using PRV HLJ as the control. The one-step growth curve indicated that the replication of resBAC-HLJ was lower than that of wild-type PRV HLJ (Figure 1G). The genome of BAC-HLJ was extracted (Szpara et al., 2011), digested, analyzed with PFGE, and compared with the genome of the parental virus PRV HLJ and pBAC-HLJ. The sizes of resBAC-HLJ and pBAC-HLJ were similar to that of PRV HLJ, and any slight differences were attributable to the substitution of genes Us7–Us8–Us9 into pBAC-GFP62 (Figure 1H).

### CRISPR/Cas9 enhances HR efficiency

Different CRISPR/Cas9s were used in single-incision CRISPR/Cas9 or double-incision CRISPR/Cas9 reactions to determine how the efficiency of HR is affected. For the single incisions, pCas9-Us2, pCas9-Us7, pCas9-Us8, and pCas9-Us9 were tested. For the double incisions, the combinations pCas9-Us7/pCas9-Us9 and pCas9-Us7/pCas9-Us8 were tested. At 72 hpi, the cells were harvested and subjected to three freeze–thaw cycles. The HR efficiencies were determined as the ratios of recombinant viral plaques to total viral plaques.

**Figure 2:**
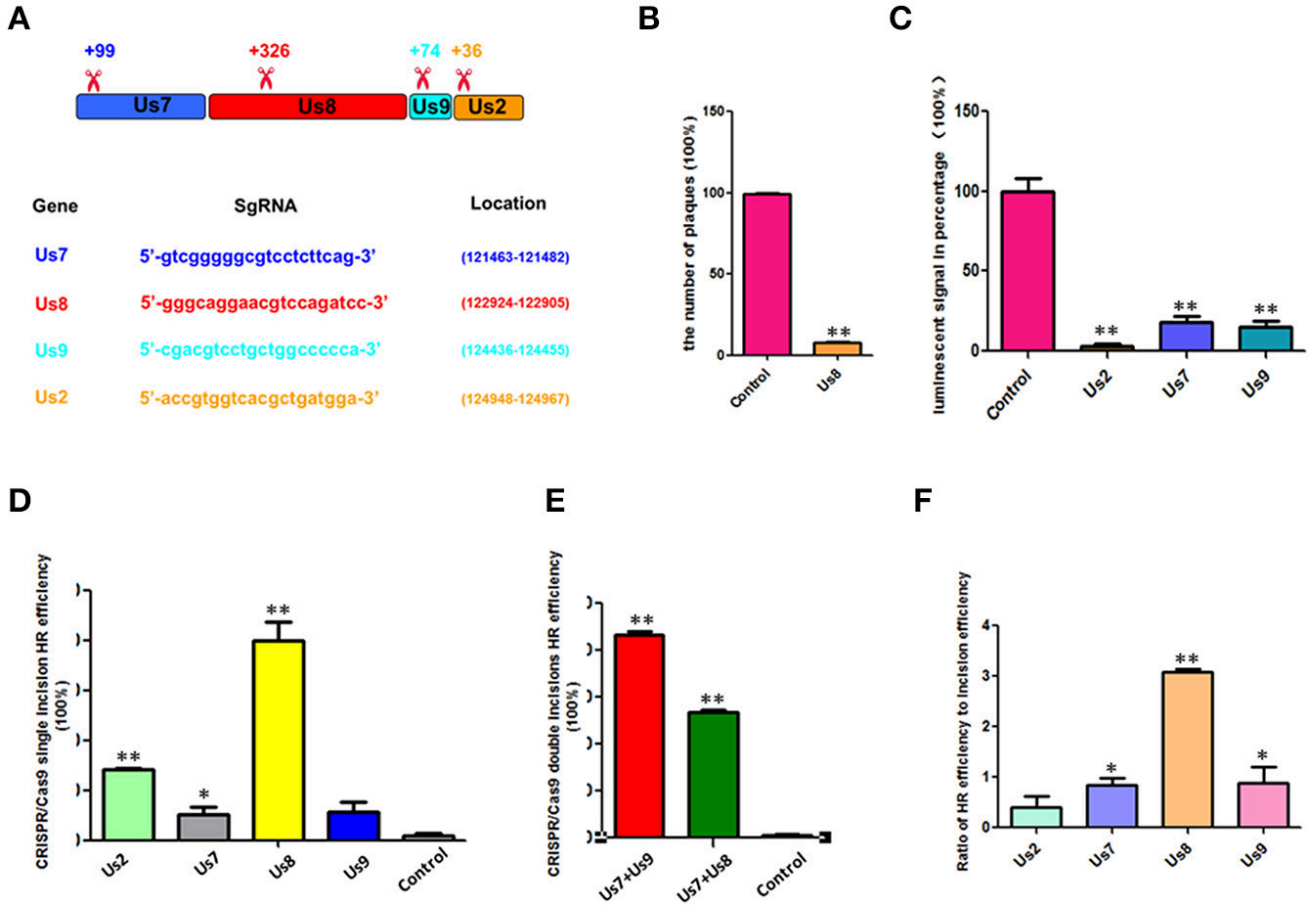
**HR efficiencies were enhanced by CRISPR/Cas9 incision. (A)** Sequences of different CRISPR/Cas9 sgRNAs, their locations, and their incision positions are shown. **(B)** pCas9-Us8 incision efficiency was determined by comparing the PRV plaque numbers with the control numbers. **(C)** Percentage of fluorescent signals for pCas9-Us2, pCas9-Us7, and pCas9-Us9 are shown by luciferase, which replaced the gene of Us8, indicating the incision efficiencies of those pCas9-sgRNAs. **(D)** HR efficiencies of Cas9 single incisions; pCas9-Us8 was up to 39%. **(E)** HR efficiencies of Cas9 double incisions. HR efficiencies of combinations pCas9-Us7/pCas9-Us9, pCas9-Us7/pCas9-Us9, and pCas9-Us7/pCas9-Us8 were all >50%, and that of pCas9-Us7/pCas9-Us9 was as high as 86%. **(F)** Ratios of HR efficiency to incision efficiency for single incisions. pCas9-Us8 had the highest ratio, pCas9-Us7 and pCas9-Us9 had similar lower ratios, and pCas9-Us2 had the lowest ratio. The data show that when the incision efficiencies were equal, pCas9-Us8 had the highest HR efficiency. ^*^P < 0.05, ^**^P < 0.01.

The sequences and locations of the sgRNAs of pCas9-Us7, pCas9-Us8, pCas9-Us9, and pCas9-Us2 are shown in Figure 2A, and their incision positions are also indicated (Figure 2A), which were usually at the start sites of their open reading frames. To determine the incision efficiency of pCas9-Us8, the plaque number was calculated and compared with the control (Figure 2B). For the other CRISPR/Cas9s, including pCas9-Us7, pCas9-Us9, and pCas9-Us2, their incision efficiencies were determined from the luminescent signals of PRV expressing firefly luciferase (Figure 2C). A lower percentage luminescent signal relative to the control signal indicated higher incision efficiency. The incision efficiency was higher for pCas9-Us2 than for pCas9-Us7 or pCas9-Us9.

The HR efficiencies of the single incisions are shown in Figure 2D. Different CRISPR/Cas9s produced different HR efficiencies. PCas9-Us8 enhanced the HR efficiency by 39%, pCas9-Us2 by 14%, and pCas9-Us7 and pCas9-Us9 by around 5%. For the double incisions, the combinations pCas9-Us7/pCas9-Us9 and pCas9-Us7/pCas9-Us8 (Figure 2E) had HR efficiencies >50%, and the former was as high as 86%. With an HR efficiency of 86%, several rounds of plaque purification, which are labor intensive and time consuming, can be avoided and the inoculum can be used directly in some future research.

Although different single incisions had different HR efficiencies, their incision efficiencies also varied. The ratios of HR efficiency to incision efficiency were determined to identify any effect of the positions of the sgRNAs (Figure 2F). PCas9-Us8 had the highest ratio; those of pCas-Us7 and pCas9-Us9 were lower and almost the same; and that of pCas9-Us2 was lowest. These results indicate that single incisions with the same incision efficiency will show more efficient HR if the incision site is adjacent to the center of the homology arms.

## Discussion

The pBAC-HLJ was rapidly constructed by applying CRISPR/Cas9 promoting HR. Apart from constructing pBAC-HLJ, another PRV virus Bartha was also subcloned into a BAC with the same transfer vector pBAC-GFP62, named pBAC-Bartha. While Bartha is a vaccine virus, which have Us8/Us9 and partial Us2 genes deleted, it is unachievable to incite these genes and we can only access to pCas-Us7. Compared to the control group, we find higher HR efficiency by the application of pCas9-Us7 (unpublished data), and this once again prove the availability of CRISPR/Cas9 as efficient knock-in tool. As double incisions of sgRNAs by CRISPR/Cas9 promote HR greatly with a linearized donor plasmid, it not only save us much time to purify a recombinant virus, but it is also possible for us to subclone the PRV isolates into BAC vector directly, which provide us the chance of studying herpesvirual versatility.

The HR efficiencies of the double incisions were higher than those of the single incisions. The combination of pCas9-Us7 and pCas9-Us9 induced HR efficiency as high as 86%. HR efficacy may be influenced by several factors. In this study, the incision efficiencies of the CRISPR/Cas9s were first identified (Tang et al., 2016), and we demonstrated that the positions of the CRISPR/Cas9 sgRNAs and their distances from the homology arms correlated with HR efficacy. For single incisions, pCas9-Us6 was also applied, but showed a lower HR efficiency even than the control group (almost one-third of the control group, unpublished data), since it not only incised PRV HLJ genome, but also incised the transfer vector pBAC-GFP62, we strongly suggest the target sites should be avoided that both incise the plasmid and genome for higher efficiency. For other sgRNAs of CRISPR/Cas9 within homology arms of single incision, we inferred that the closer the incision site is to the center of the homology arms, the higher is the HR efficiency, and this result is consistent with the effects of Cas9-mediated nicks on HR efficiency (Ran et al., 2013). Double incisions have been done by pCas9-Us6/pCas9-Us2 (unpublished data), pCas9-Us7/pCas9-Us9 and pCas9-Us7/pCas9-Us8. Of the viruses without GFP, we found 150 viruses in average in 10^−3^ dilution by pCas9-Us7/pCas9-Us8, and 13 viruses in average in 10^−3^ dilution by pCas9-Us7/pCas9-Us9, and we failed to detect any virus (including the recombinant virus and wild type virus) by pCas9-Us6/pCas9-Us2 even in 10^−1^ dilution, which we suppose that the larger the distances between two incision sites, the harder the virus to be recovered by NHEJ. A similar work has been done by Tang et al, who acquired the recombinant virus almost 100% with genes Us7/Us8/Us9 deleted by pCas9-Us7 and pCas9-Us2 (unpublished data), which again support the speculation that more damage are induced by double incisions with longer distances between them. A comparison of HR efficiencies by pCas9-Us7/pCas9-Us9 and pCas9-Us7/pCas9-Us8 showed that while incision sites are within homology arms, the more two incision sites near the homology arms, the higher the HR efficiency would be. For us, the best choice of promoting HR is by double incisions and that the two incision sites near and within the homology arms but pCas9 gRNAs do not incise them.

In this study, we showed that the combination of linearized donor plasmid and double incisions of CRISPR/Cas9 that achieved best HR in PRV. Double incisions are more efficient than single incision, we suppose double incised viruses are less easily to be recovered by NHEJ, in which it may replicates faster than the recombinant virus and dilutes the recombinant virus. While a circular donor plasmid Tang-Luc-EGFP-HR vector (Tang et al., 2016) was co-transfected with PRV genome and by single incision of pCas9-Us8 as well as double incisions of pCas9-Us7 and pCas9-Us9 at the same amount, the single incision and double incisions both reach an HR efficiency of 0.38 percent. We suppose the reason may be the formation of superhelix of circular donor plasmid, and thus difficult to be incised by CRISPR/Cas9. However, in the recent construction of an Epstein–Barr virus-BAC system, only a circular donor plasmid, rather than a linearized one, produced successful HR though without exact HR efficiency (Kanda et al., 2016). Another study in which the PRV genome was manipulated with CRISPR/Cas9 showed that HR failed when a linearized homologous repair donor plasmid was inserted into the PRV genome, but achieved a recombinant efficiency of almost 50% when a homology-independent DNA repair mechanism was used (Liang et al., 2016). The different results generated with linearized donor plasmids and circular donor plasmids and the efficiencies of HR and nonhomologs recombination must be explored further.

We also think the length of homology arms will influence the HR efficiency. As paper published by Li et al, they conclude that increasing the homology arm sizes enhanced HR efficiency (Li et al., 2014). Beumer showed us that a comparison of different homology arm sizes induced different HR efficiencies, and the length of ~2 and ~4 kb have higher efficiencies than length of 1 or 0.5 kb homology arms, which might provide us a relationship between the length of homology arms and HR efficiencies (Beumer et al., 2013). So from our perspective, we decide the homology arms of ~1.5 to ~2 kb are better to induce high HR.

While we suppose that different cell lines may be varied in response to the incisions of CRISPR/Cas9 and leading to different HR efficiencies, further research need to be done to explore the differences and the relative mechanism. For different viruses, though we have only tested the CRISPR/Cas9 in PRV, many papers published online already show that the CRISPR/Cas9 have ability and high efficiency in both knocking in and knocking out different viruses (such as HSV, EBV, HIV). Future research will be directed toward unraveling the other factors affecting HR efficiency mediated by CRISPR/Cas9, the precise mechanisms that enhance HR efficiency, and the application of CRISPR/Cas9 to other viruses. This will, in turn, widen our knowledge of CRISPR/Cas9 as an efficient gene knock-in tool and clarify the potential of CRISPR/Cas9 in promoting HR as a specialized instrument. Though the CRISPR/Cas9 is efficient in knocking-in genes, it still has limitations. While mutations in essential genes may lead to death of the virus, the CRISPR/Cas9 can only be applied to insert genes in nonessential parts of the genome, which is a drawback shared by other technologies promoting HR.

## Conclusions

We have demonstrated that BAC construction with large-genome viruses can be achieved in a simple rapid way with highly efficient CRISPR/Cas9-mediated HR. HR efficiency is enhanced when CRISPR/Cas9 is performed with a linearized donor plasmid, and the positions of the incisions and their distance from the homologous arms affect the efficiency of HR. Double incisions produce higher HR efficiency than single incisions, and we achieved a HR efficiency by up to 86%, the highest ever reported for PRV recombination.

## Author contributions

All authors listed, have made substantial, direct and intellectual contribution to the work, and approved it for publication.

### Conflict of interest statement

The authors declare that the research was conducted in the absence of any commercial or financial relationships that could be construed as a potential conflict of interest.
