# Supplementary material for: Highly Efficient CRISPR/Cas9-Mediated Homologous Recombination Promotes the Rapid Generation of Bacterial Artificial Chromosomes of Pseudorabies Virus
Source: Front Microbiol. 2016 Dec 23;7:2110. doi: 10.3389/fmicb.2016.02110 (PMC5179515; doi:10.3389/fmicb.2016.02110)

**SUPPLEMENTAL MATERIAL**

**Fig S1 A graphical abstract of the experiment procedure.** At first, the pBeloBAC11 was inserted by two loxp fragments, which generated pBACloxp. Then a gene expressing GFP was inserted and generated pBAC-GFP, by amplifying the upper homology arm Us6 and the lower homology arm Us2 and inserted into pBAC-GFP, the pBAC-GFP62 was generated and linearized. Then PRV HLJ genome and the transfer vector pBAC-GFP62 was transfected to cells, meanwhile a series of sgRNAs of CRISPR/Cas9 was applied to test their role in promoting HR. After acquiring the purified recombinant virus, it was electroporated to DH10B E.coli and generated pBAC-HLJ, then the pBAC-HLJ was transfected to rescue the virus. Finally is test the resBAC-HLJ from various aspects.


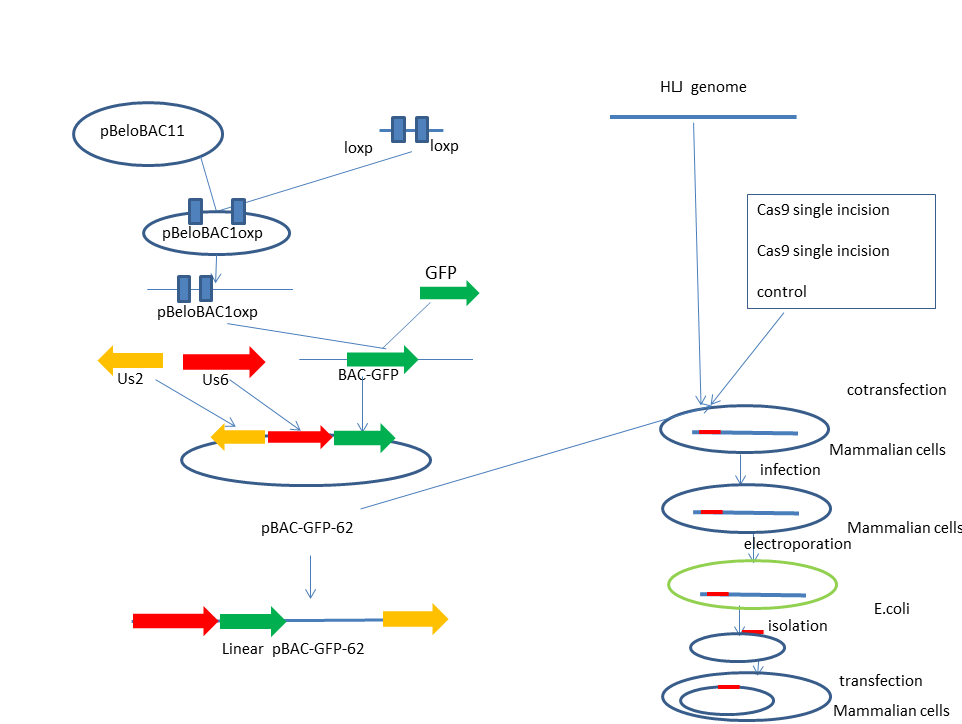

Supplement: Supplementary file 1 [file DataSheet1.DOCX]
