# Supplementary material for: Highly Efficient CRISPR/Cas9-Mediated Homologous Recombination Promotes the Rapid Generation of Bacterial Artificial Chromosomes of Pseudorabies Virus
Source: Front Microbiol. 2016 Dec 23;7:2110. doi: 10.3389/fmicb.2016.02110 (PMC5179515; doi:10.3389/fmicb.2016.02110)
Supplement: Supplementary file 2 [file DataSheet2.DOCX]

**SUPPLEMENTAL MATERIAL**

**Figure S2. Identification of resBAC-HLJ by PCR.** Different portions of resBAC-HLJ genome were tested through PCR to verify whether the full genome of resBAC-HLJ was inserted into the BAC. They included BAC, GFP, gB, gC, gG, gH, gK, gN. The results revealed that of the genes tested were existed. Also we test gI and gE which we have deleted in resBAC-HLJ with PRV HLJ as a contrast. Results showed that the resBAC-HLJ failed to detect gI and gE while they all existed in PRV HLJ.
